# MiR-34a/c-Dependent PDGFR-α/β Downregulation Inhibits Tumorigenesis and Enhances TRAIL-Induced Apoptosis in Lung Cancer

**DOI:** 10.1371/journal.pone.0067581

**Published:** 2013-06-21

**Authors:** Michela Garofalo, Young-Jun Jeon, Gerard J. Nuovo, Justin Middleton, Paola Secchiero, Pooja Joshi, Hansjuerg Alder, Natalya Nazaryan, Gianpiero Di Leva, Giulia Romano, Melissa Crawford, Patrick Nana-Sinkam, Carlo M. Croce

**Affiliations:** 1 Department of Molecular Virology, Immunology and Medical Genetics, Comprehensive Cancer Center, the Ohio State University, Columbus, Ohio, United States of America; 2 Phylogeny, Inc., Columbus, Ohio, United States of America; 3 Department of Morphology and Embryology, Human Anatomy Section, University of Ferrara, Ferrara, Italy; 4 Pulmonary, Allergy, Critical Care and Sleep Medicine, The Ohio State University Comprehensive Cancer Center, Columbus, Ohio, United States of America; Institute of Medical Science, University of Tokyo, Japan

## Abstract

Lung cancer is the leading cause of cancer mortality in the world today. Although some advances in lung cancer therapy have been made, patient survival is still poor. MicroRNAs (miRNAs) can act as oncogenes or tumor-suppressor genes in human malignancy. The miR-34 family consists of tumor-suppressive miRNAs, and its reduced expression has been reported in various cancers, including non-small cell lung cancer (NSCLC). In this study, we found that miR-34a and miR-34c target platelet-derived growth factor receptor alpha and beta (PDGFR-α and PDGFR-β), cell surface tyrosine kinase receptors that induce proliferation, migration and invasion in cancer. MiR-34a and miR-34c were downregulated in lung tumors compared to normal tissues. Moreover, we identified an inverse correlation between PDGFR-α/β and miR-34a/c expression in lung tumor samples. Finally, miR-34a/c overexpression or downregulation of PDGFR-α/β by siRNAs, strongly augmented the response to TNF-related apoptosis inducing ligand (TRAIL) while reducing migratory and invasive capacity of NSCLC cells.

## Introduction

Lung cancer is the most common cause of cancer death worldwide [1]. Despite years of research, the prognosis for patients with lung cancer remains dismal. The most frequent type, non-small cell lung cancer (NSCLC) (85%), shows an overall five year survival of 15%. Isoforms of platelet-derived growth factor receptor (PDGFR) and its ligand, PDGF, constitute a family of receptors and ligands involved in proliferative and prosurvival signaling within cells. The PDGFR/PDGF system includes two receptors (PDGFR-α and PDGFR-β) and four ligands (PDGFA, PDGFB, PDGFC, and PDGFD). Ligand binding induces receptor dimerization, enabling autophosphorylation of specific tyrosine residues and subsequent recruitment of a variety of signal transduction molecules. PDGFR regulates normal cellular growth and differentiation [2] and expression of activated PDGFR promotes oncogenic transformation [3]. Numerous *in vitro* and *in vivo* studies showed that inhibition of PDGFR-α signaling disrupts cancer cell survival in the subset of gastrointestinal stromal tumors (GISTs) with activating PDGFR-α mutations [4,5]. In a recent study of 150 NSCLC patient samples, activated PDGFR-α was detected in 13% of cases [6], suggesting that a subset of these patients might benefit from therapies directed against PDGFR-α. Moreover, PDGFR-α overexpression has been observed in metastatic versus nonmetastatic medulloblastoma patient samples and disrupting PDGFR-α function inhibited the metastatic potential of medulloblastoma cells in vitro [7]. Given its progrowth role in cell signaling, PDGFR-α has become an attractive therapeutic target in a number of human malignancies. In non–small cell lung cancer, cytoplasmic PDGFR-α expression by tumor is a negative prognostic indicator [8], confirming that the PDGF axis may be biologically relevant. All members of the PDGF family display potent angiogenic activity *in vivo*, and from this point of view, PDGF-B/PDGFRβ axis was the most extensive evaluated. In the null mice it was shown that PDGF-B and PDGFRβ are critically involved in vascular development. The role of PDGF/PDGFR in vascular development is supported by knockout experiments [9,10]. MicroRNAs (miRNAs), a class of ~ 22 nt endogenous RNAs, are important regulators of gene expression and have been implicated in the regulation of critical processes that are deregulated in cancer cells, as proliferation [11] differentiation [12] and apoptosis [13]. Alterations in miRNA expression in cancer have been documented in numerous studies and suggest that miRNAs critically contribute to the cancer cell phenotype [14,15]. Furthermore, some miRNA-encoding genes have been classified as oncogenic or tumor suppressive genes according to their function in cellular transformation and altered expression in tumors.

In 2007, reports from several laboratories showed that members of the miR-34 family are direct p53 targets, and that their upregulation induced apoptosis and cell-cycle arrest [16,17]. In mammalians, the miR-34 family comprises three processed miRNAs that are encoded by two different genes: miR-34a is encoded by its own transcript, whereas miR-34b and miR-34c share a common primary transcript. Moreover, the promoter region of miR-34a, miR-34b and miR-34c contains CpG islands and aberrant CpG methylation reduces miR-34 family expression in multiple types of cancer [18,19,20].

In this study, we show that miR-34a and miR-34c, are strongly downregulated in NSCLC cells and lung tumors whereas they are highly expressed in normal lung tissues. Moreover, miR-34a and miR-34c, by targeting PDGFR-α and PDGFR-β, increase TRAIL-induced apoptosis and decrease invasiveness of lung cancer cells. Furthermore, our results suggest, for the first time, that combinatory treatment of TRAIL and PDGFR inhibitors could be effective for anti-NSCLC therapy.

## Results

### MiR-34a and miR-34c target PDGFR-α and PDGFR-β 3’ UTRs

**Figure 1:**
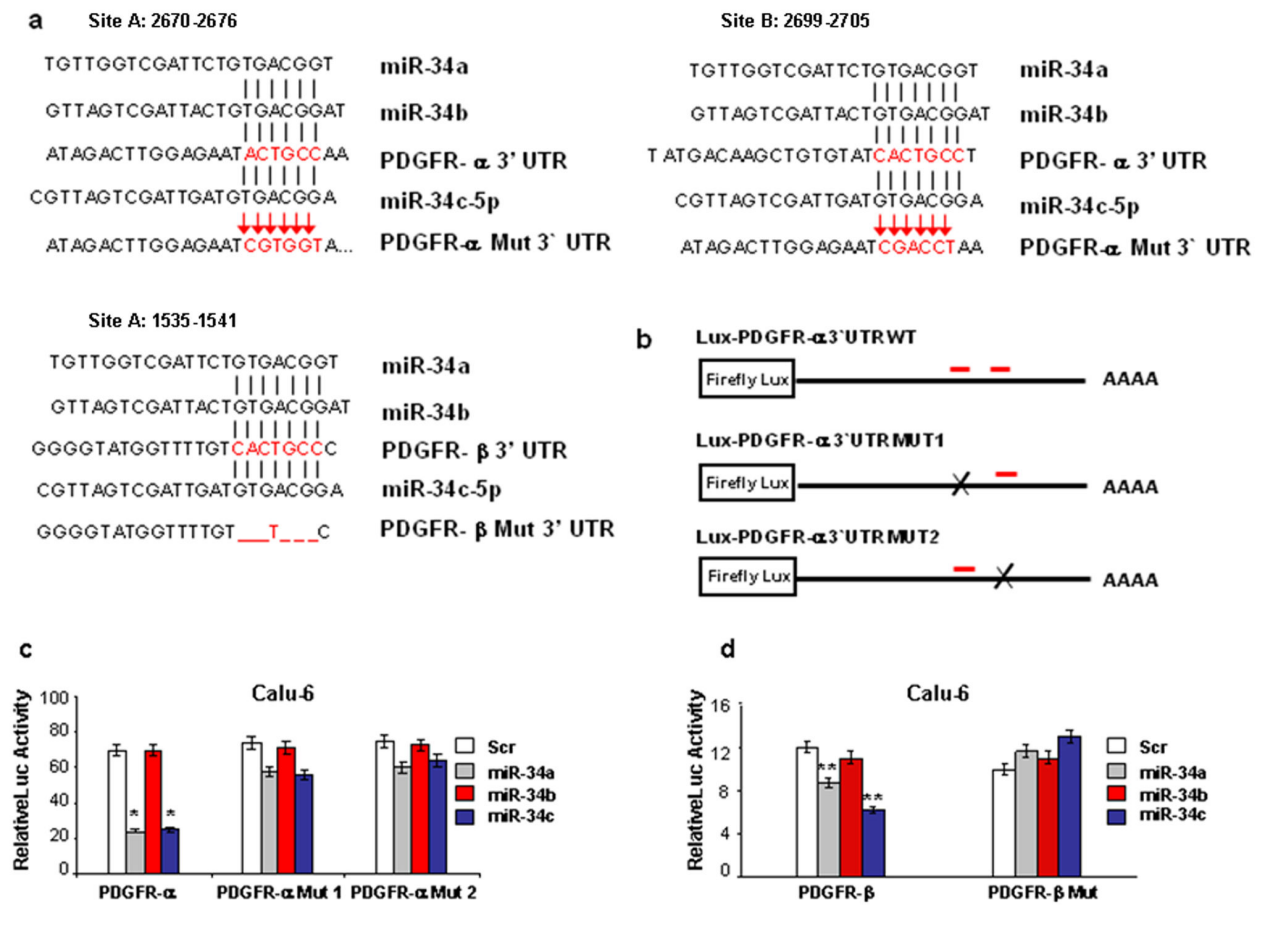
MiR-34a and miR-34c target PDGFR-α and PDGFR-β 3’ UTRs. (**a**) PDGFR-α and PDGFR-β 3’ UTRs contain, respectively, two and one predicted miR-34a, -34b and -34c binding sites. In the figure the alignment of the seed regions of miR-34a/c with PDGFR-α and PDGFR-β 3’ UTRs is shown. The sites of target mutagenesis are indicated in red. _deleted nucleotides. (**b**) PGL3 control-PDGFR-α constructs containing two PDGFR-α binding sites (in red). Deletion of one of the two PDGFR-α sites was used to generate the mutant luciferase palsmids. (**c**–**d**) PDGFR-α and PDGFR-β 3’ UTRs are direct targets of miR-34a and miR-34c. pGL3-PDGFR-α and pGL3-PDGFR-β luciferase constructs, containing a wild-type (left side of the histograms) or mutated (right side of the histograms) PDGFR-α and PDGFR-β 3’ UTRs, were transfected into Calu-6 cells. Relative repression of firefly luciferase expression was standardized to a transfection control. The reporter assays were performed three times with essentially identical results. *P<0.0001, **P<0.05 by two tailed Student’s t test.

Among the miRNAs, miR-34 family members play important tumor suppressive roles, as they are directly regulated by p53 and compose the p53 network [16,17]. A previous study indicated that miR-34 methylation was present in NSCLC and was significantly related to an unfavorable clinical outcome [20]. First, we analyzed by Real Time PCR (qRT-PCR) miR-34a, -34b and -34c expression in 5 different NSCLC cell lines with p53 WT, mutant or null (Figure S1a in File S1). MiR-34a, -34b and -34c had low or absent expression in all the five cell lines (Figure S1b in File S1). To identify miR-34a, -34b, and -34c targets, we performed a bioinformatics search (Targetscan, Pictar) for putative mRNA targets. Among the candidate targets, 3’ UTR of human PDGFR-α and PDGFR-β contained regions (PDGFR-α nucleotides 2670–2676; 2699-2705; PDGFR-β nucleotides 1535-1541) that matched the seed sequences of hsa-miR-34a, -34b and -34c (Figure 1a). PDGFR-α and PDGFR-β have been reported to be overexpressed and related to poor outcome in lung cancer [21]. To verify whether PDGFR-α and PDGFR-β were direct targets of miR-34a, -34b and -34c, PDGFR-α 3’ UTR, containing two miR-34a, -34b and -34c binding sites and PDGFR-β 3’ UTR, containing one miR-34a, -34b and -34c binding site (Figure 1a,b), were cloned downstream of the luciferase open reading frame. Interestingly, increased expression of miR-34a and miR-34c, and not miR-34b, upon transfection, confirmed by qRT-PCR (data not shown), significantly decreased luciferase activity, indicating a direct interaction between the miRNAs and PDGFRα and PDGFRβ 3’ UTRs (Figure 1c,d). Target gene repression was rescued by mutations in the complementary seed sites (Figure 1b,c,d). Taken together the results indicate that miR-34a and miR-34c and not miR-34b directly target PDGFR-α and PDGFR-β.

### MiR-34a and miR-34c are inversely related to PDGFR-α/β expression *in vitro* and *in vivo*

**Figure 2:**
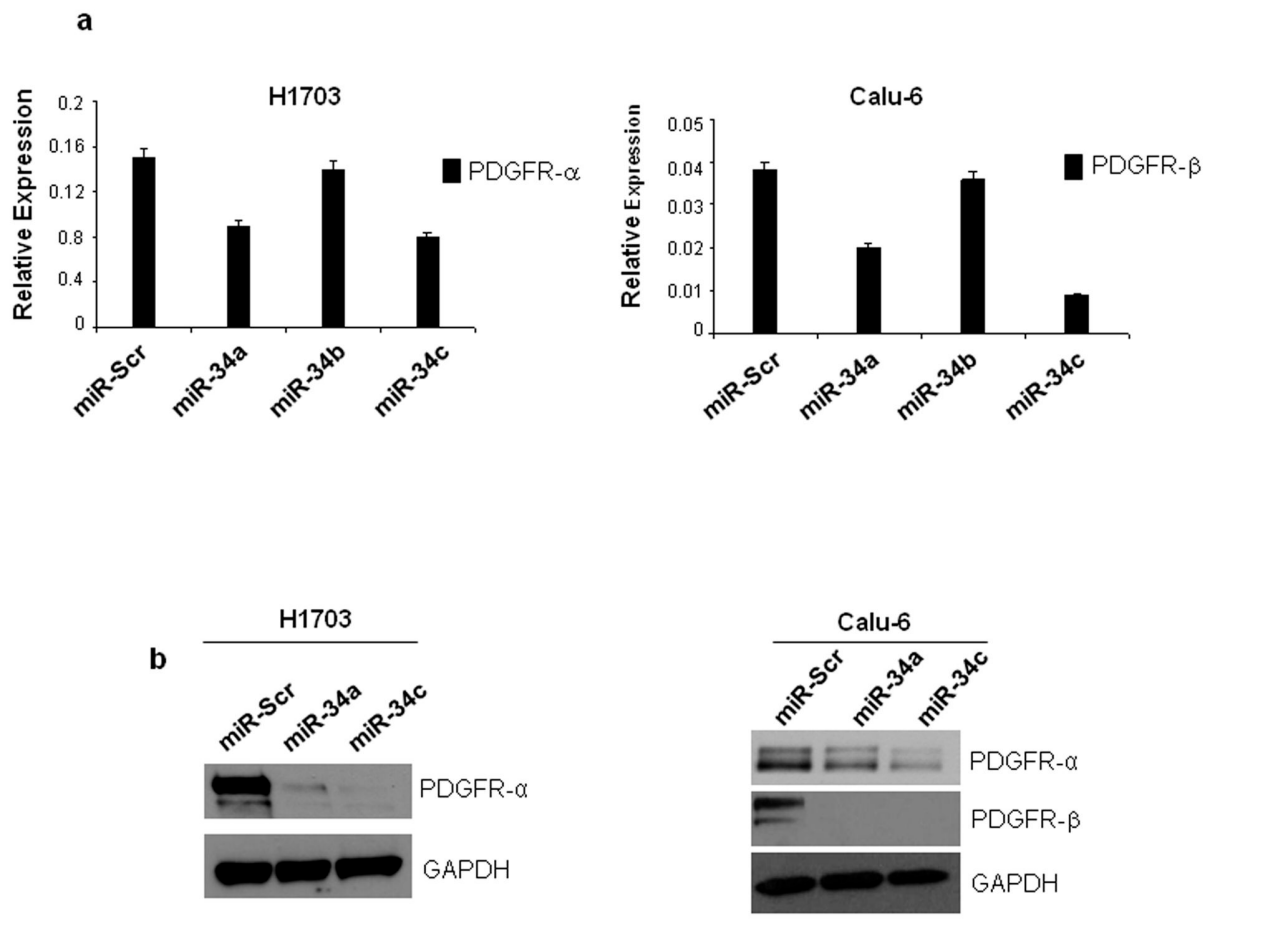
MiR-34a and miR-34c reduce PDGFR-α and PDGFR-β mRNA and protein levels. (**a**) qRT-PCR showing PDGFR-α and PDGFR-β mRNAs downregulation in Calu-6 and H1703 cells after miR-34 and miR-34c but not miR-34b enforced expression (**b**) miR-34a and miR-34c enforced expression decreases endogenous levels of PDGFR-α/β protein levels in H1703 and Calu-6 NSCLC. Cells were transfected with either scrambled, miR-34a or miR-34c for 72h. PDGFR-α and PDGFR-β expression was assessed by western blot. Loading control was obtained using GAPDH antibody. *P<0.05, **P<0.001 by two tailed Student’s t test.

Next, we analyzed the consequences of the ectopic expression of miR-34a and -34c in Calu-6 and H1703 cells. We chose these two cell lines because of the high expression levels of PDGFR-α (H1703 and Calu-6) and PDGFR-β (Calu-6) (data not shown). Increased expression of miR-34a and miR-34c upon transfection was confirmed by qRT-PCR (data not shown) and then the effects on PDGFR-α and PDGFR-β mRNA and protein levels were analyzed by qRT-PCR and western blot. MiR-34a and -34c (and not miR-34b) overexpression significantly reduced PDGFR-α and PDGFR-β mRNAs (Figure 2a) and the endogenous protein levels, compared to the cells transfected with a scrambled pre-miR (Figure 2b). The expression levels of the four PDGF ligands (PDGFA, PDGFB, PDGFC, PDGFD) after miR-34a and miR-34c enforced expression were also evaluated in both Calu-6 and H1703 cells. PDGFD was barely expressed in both cell lines; we did not find any variation of the expression of PDGFA, PDGFB and PDGFC (data not shown). In summary, these results supported the bioinformatics predictions indicating PDGFR-α and PDGFR-β 3’ UTRs as targets of miR-34a and -34c.

**Figure 3:**
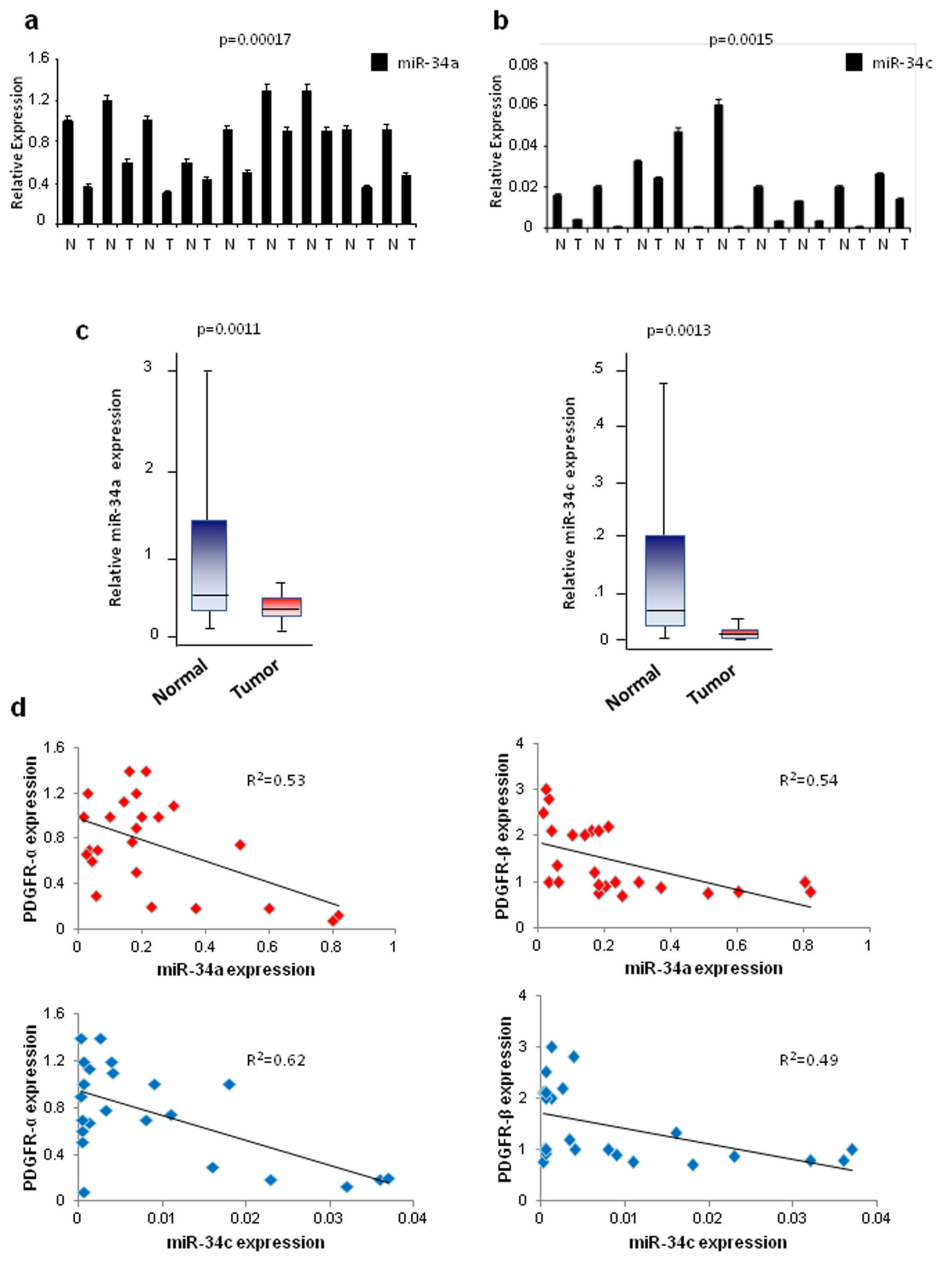
MiR-34a/c and PDGFR-α/β are inversely correlated in normal and tumor lung tissue samples. (**a**–**b**) qRT-PCR on 18 lung tumor and normal tissues. MiR-34a and miR-34c are downregulated in the tumors compared to the normal lung tissues. (**c**) Box plots showing miR-34a and miR-34c expression in 48 lung normal and cancer tissues. (**d**) XY scatter plots showing inverse correlation between miR-34a/c and PDGFR-α/β. Two-tailed Student’s t test was used to verify the significance. P<0.05.

To verify the downregulation of miR-34a and -34c also *in vivo*, 9 lung tumors (among adenocarcinoma and squamous cell carcinoma) and the adjacent histologically normal lung tissues were analyzed for miR-34a and -34c expression. As shown in Figure 3a and b, miR-34a and miR-34c expression was lower in the tumor compared to the normal samples (Figure 3a,b).

**Figure 4:**
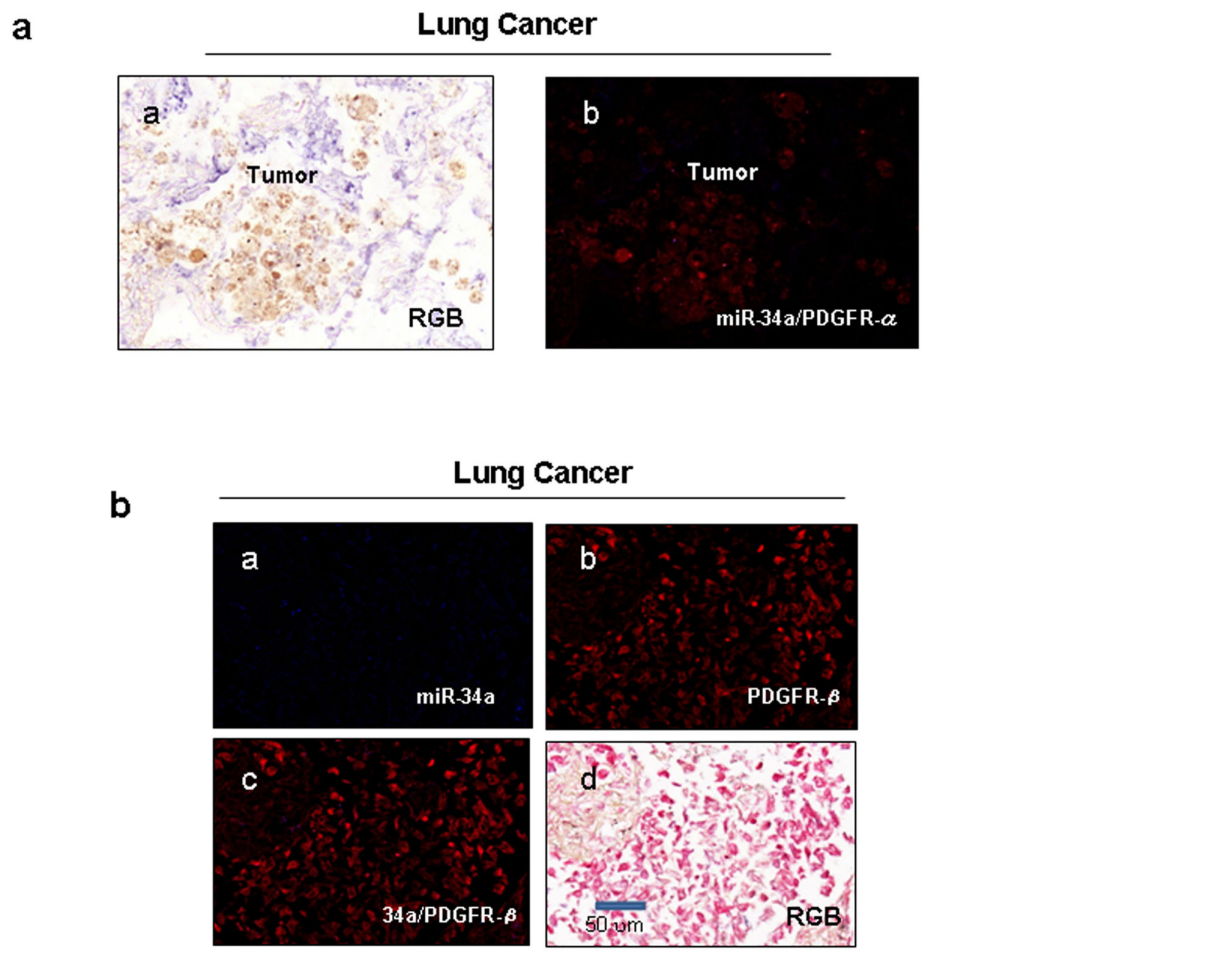
MiR-34a/c and PDGFR-α/β co-expression *in vivo*. (**a**–**b**) Immunohistochemistry and in situ hybridization on 107 lung cancer tissues samples. MiR-34a (blue) and PDGFR-α/β (brown/red, respectively in RGB and each fluorescent red in Nuance converted image) expression was inversely related in lung cancers and the adjacent normal lung tissues. These serial sections were analyzed for miR-34a expression by in situ hybridization, followed by immunohistochemistry for PDGFR-α/β. (**a**) Representative example: Co-expression analysis of miR-34a and PDGFR-α. Note lack of expression in the merged image (panel b) (fluorescent yellow = co-expression). (**b**) Representative example: miR-34a= blue (panel a), PDGFR-β=red (panel b), co-expression= yellow (panel c). RGB= Regular Green Blue image of the ISH/lHC reaction shown in panels a-c. Scale bar indicates 50 μm. The magnification is the same for all the panels.

Moreover, we analyzed miR-34a, 34c and PDGFR-α/β mRNA expression in 24 primary human lung tumor specimens in comparison with 24 normal tissues. MiR-34a and -34c were almost undetectable in the tumor and highly expressed in the normal lung samples tested (Figure 3c). Remarkably, an inverse correlation between miR-34a/c and PDGFR-α and PDGFR-β was observed (Figure 3d). To further corroborate these findings, in situ hybridization (ISH) analysis was performed using 5’-dig-labeled LNA probes on lung tumors and normal tissues, followed by immunohistochemistry (IHC) for PDGFR-α and PDGFRβ. Most lung cancer cells showed low expression of miR-34a and high expression of PDGFR-α/β, whereas the adjacent non-malignant lung expressed PDGFR-α rarely and abundantly expressed miR-34a. MiR-34a and PDGFR-α/β expression in the majority of the 107 different tumors analyzed was basically mutually exclusive (Figure 4a,b and Figure S2 in File S1).

### MiR-34a and miR-34c overcome TRAIL resistance of NSCLC cells through PDGFR-α and PDGFR-β downregulation

**Figure 5:**
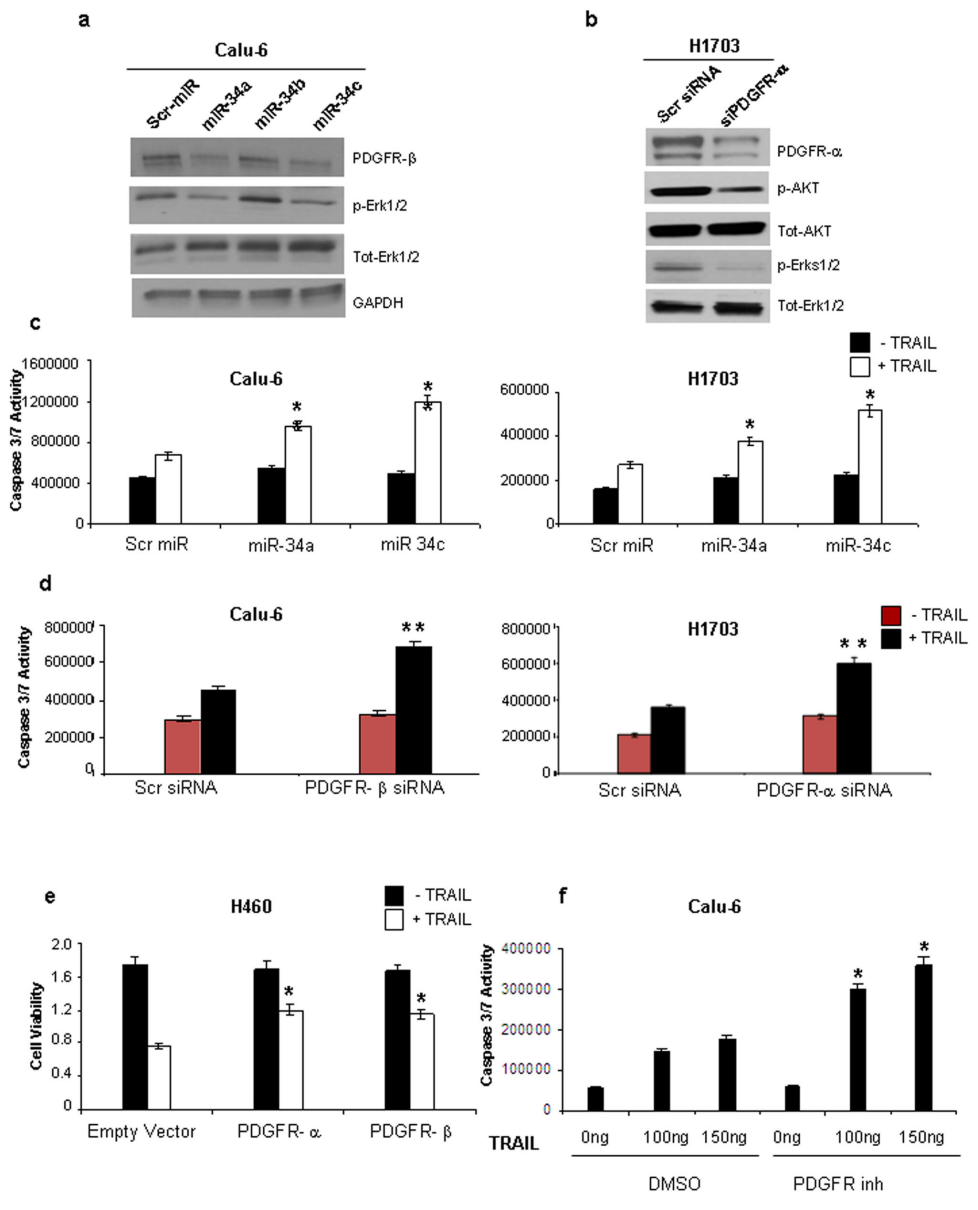
MiR-34a and miR-34c overexpression or PDGFR-α/β silencing increases the response of NSCLC cells to TRAIL-induced apoptosis. (**a**) Western blot in Calu-6 cells after miR-34a, -34b and -34c forced expression. MiR-34a or miR-34c and not miR-34b forced expression decreases PDGFRβ expression levels and reduces the activation of the ERK1/2. (**b**) Western blot showing the inactivation of the Akt and ERKs pathways after PDGFR-α silencing. (**c**) Caspase 3/7 assay. MiR-34a and -34c enforced expression in Calu-6 and H1703 semi-resistant cells, increases the response to TRAIL-induced apoptosis. (**d**) Caspase 3/7 assay showing that PDGFR-α or PDGFR-β silencing increases the response to TRAIL-induced apoptosis. (**e**) PDGFR-α or PDGFR-β overexpression in H460 TRAIL-sensitive cells decreases the response to the drug. (**f**) Combined treatment of PDGFR inhibitor (20 μM) and different TRAIL concentrations (0-100-150 ng/ml) for 24h sensitizes NSCLC cells to TRAIL-induced apoptosis. *P<0.001, ** P<0.05.

TNF-related apoptosis-inducing ligand (TRAIL) is a member of the tumor necrosis factor family, known to induce apoptosis in a variety of different tumor types. TRAIL is able to specifically induce cell death in cancer cells while sparing normal cells and is currently being tested as a promising anti-tumor agent in clinical trials [22]. However, many tumors including NSCLC are resistant to TRAIL thus limiting its therapeutic application. Since PDGFR-α and PDGFR-β regulate the PI3K/Akt and ERK1/2 pathways [23,24,25], we next examined, by immunostaining, the expression and/or activation of some of the proteins involved in these pathways following miR-34a and miR-34c enforced expression or PDGFR-α/β silencing by siRNAs. As shown in Figure 5a, phosphorylation levels of ERKs decreased after miR-34a and miR-34c enforced expression compared to cells transfected with the control miR. PDGFR-α silencing reduced the activation of both Akt and ERK1/2 (Figure 5b). We previously demonstrated that the PI3K/AKT pathway plays a key role in TRAIL-induced apoptosis [26], therefore the effects of miR-34a and miR-34c overexpression on cell survival and TRAIL resistance of NSCLC were examined. First, we performed a proliferation assay on Calu-6 and H1703 TRAIL semi-resistant cells after enforced expression of miR-34a and miR-34c. 48h after transfection cells were exposed to TRAIL for 24h and then cell proliferation was assessed using an MTT assay. Calu-6 and H1703 cells overexpressing miR-34a and -34c showed a significant lower proliferation rate compared to the control cells (Figure S3a in File S1). Moreover, caspase 3/7 assay revealed an increase in TRAIL sensitivity after miR-34a and -34c enforced expression or PDGFR-α/β silencing, compared to the cells transfected with a scrambled miR or siRNA (Figure 5c,d).

Furthermore, overexpression of PDGFR-α and PDGFR-β in H460 TRAIL-sensitive cells, increased the resistance to the drug (Figure 5e and Figure S3c in File S1). Conversely, treatment of Calu-6 cells with a PDGFR inhibitor significantly increased the sensitivity to TRAIL-induced apoptosis (Figure 5f and Figure S3d in File S1). Intriguingly, overexpression of PDGFR-α or PDGFR-β (using two plasmids containing only the coding sequences and not the 3’ UTRs of PDGFR-α/β) along with miR-34a or miR-34c, decreased the sensitivity to TRAIL-induced apoptosis, as assessed by both MTT and caspase 3/7 assay (Figure S4 in File S1). The results suggest that PDGFR-α and PDGFR-β play an important role in TRAIL-induced apoptosis and that PDGFR inhibitor can sensitize NSCLC cells to TRAIL with important therapeutic consequences.

### PDGFR-α/β downregulation by miR-34a and miR-34c inhibits migration and invasiveness of NSCLC cells

**Figure 6:**
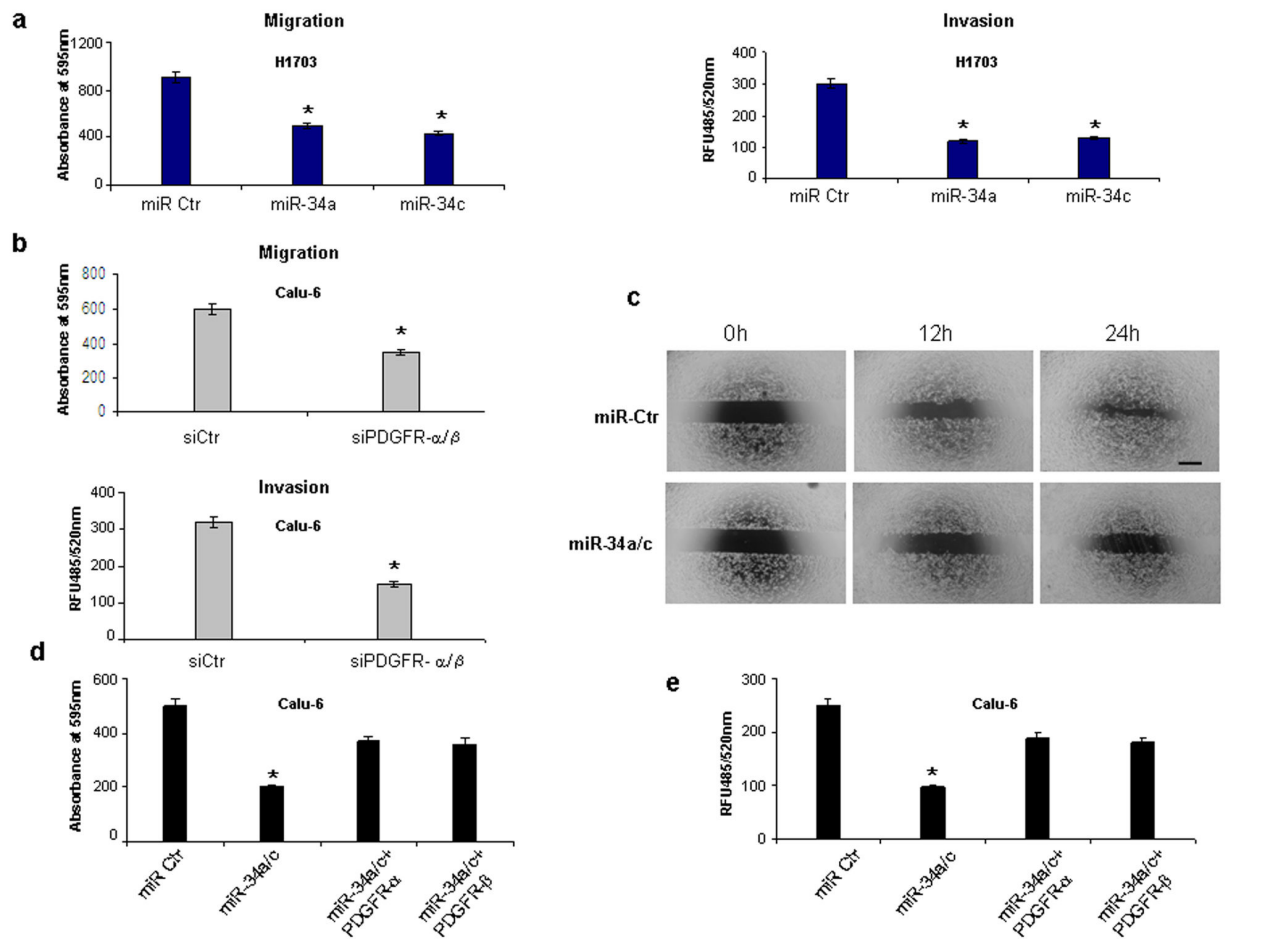
MiR-34a and miR-34c overexpression or PDGFR-α/β silencing decreases migratory and invasive capacity of NSCLC cells. (**a**) MiR-34a and -34c enforced expression reduces migratory and invasive capabilities of H1703 cells. (**b**) PDGFR-α and PDGFR-β silencing reduces migratory and invasive capabilities of Calu-6 cells. RFU= Relative Fluorescence Units. (**c**) Representative photographs of scratched areas of the confluent monolayer of Calu-6 cells transfected with miR-34a/c or control miRNA (miR-Ctr) at 0h, 12h and 24h after wounding with a pipet tip. Scale bar, 100 μm. The magnification is the same for all the panels. (**d**–**e**) PDGFR-α and PDGFR-β overexpression partially rescues migratory and invasive capabilities of Calu-6 cells. * P<0.05.

Because PDGFR-α/β regulate the PI3K/AKT pathway, notably involved in migration and invasion of different tumors [27,28], we investigated if miR-34a/c could influence NSCLC migration and invasion through PDGFR-α and PDGFR-β downregulation. To directly test the functional role of miR-34a/c in tumorigenesis, we overexpressed these two miRNAs or silenced PDGFR-α/β in Calu-6 or H1703 cells. Intriguingly, we observed a significant decrease of the migratory and invasive capabilities of Calu-6 and H1703 cells after miR-34a or miR-34c overexpression (Figure 6a) as well after PDGFR-α and PDGFR-β downregulation (Figure 6b), confirmed also by scratch-wound assay (Figure 6c). To further verify that PDGFR-α and PDGFR–β were involved in tumorigenesis of NSCLC cells, miR-34a and -34c were transfected in Calu-6 cells alone or in combination with a plasmid overexpressing only the coding sequence and not the 3’ UTR of PDGFR-α and PDGFR-β. MiR-34a/c enforced expression reduced migration and invasion of Calu-6 cells but overexpression of PDGFR-α or PDGFR-β, along with the two microRNAs, partially restored the migration and invasion capabilities, suggesting that miR-34a/c regulate NSCLC tumorigenesis, at least in part, through PDGFR-α/ β (Figure 6 d,e).

## Discussion

Lung cancer is the leading cause of cancer death in both men and women worldwide^1^. The American cancer Society estimates 156,940 deaths from lung cancer in the United States for 2011 alone [29]. Non-small cell lung cancer (NSCLC) accounts for the majority of all lung cancer cases and is a leading cause of cancer mortality [30].

The high mortality rate associated with lung cancer has prompted numerous exhaustive efforts to identify novel therapeutic targets and treatment modalities for this deadly disease. Platelet-derived growth factor receptors (PDGFRs) and their ligands, platelet-derived growth factors (PDGFs) play critical roles in mesenchymal cell migration and proliferation. Abnormalities of PDGFR/PDGF are thought to contribute to a number of human diseases and especially malignancy [31].

MicroRNAs are small noncoding RNAs that show deregulation in most cancers. There is growing evidence that they play substantial roles in the pathogenesis and prognosis of human malignancies and in the resistance to chemotherapeutic drugs [32,33]. Tumor necrosis factor-related apoptosis-inducing ligand (TRAIL) triggers apoptosis in tumor cells, but when used alone, it is ineffective at treating TRAIL-resistant tumors. This resistance is challenging for TRAIL-based anti-cancer therapies. In this study, we found that miR-34a and miR-34c are strongly downmodulated in both NSCLC cells and lung tumors compared to normal tissues. Enforced expression of miR-34a and miR-34c downregulated PDGFR-α and PDGFR-β mRNA and protein levels. Luciferase and western blot experiments demonstrated that PDGFR-α and PDGFR-β are direct targets of miR-34a and miR-34c but not of miR-34b. The resistance of many types of cancer to conventional chemotherapies is a major factor undermining successful cancer treatment. AKT activation also contributes to tumorigenesis and tumor metastasis, and as shown most recently, resistance to chemotherapy [26,34]. As a result, both *in vitro* and *in vivo* studies combining small molecule inhibitors of the PI3K/Akt pathway with standard chemotherapy have proven successful in attenuating chemotherapeutic resistance. Specifically, inhibiting AKT activity may be a valid approach to treat cancer and increase the efficacy of chemotherapy.

Protein kinases are major regulators of most cellular signaling pathways. Among them, receptor tyrosine kinases (RTKs), such as PDGFR, play pivotal roles in promoting cellular growth and proliferation by transducing extracellular stimuli to intracellular signaling circuits [35]. A prominent component of the intracellular signaling machinery is the PI3K/Akt(PKB)/mammalian target of rapamycin (PI3K/Akt[PKB]/mTOR) pathway [36,37]. Aberrant activation of this pathway by mutation of any of multiple genes is known to occur in the majority of human cancers through various mechanisms [38,39]. In a previous work [26], we demonstrated that MET, through the activation of the PI3K/AKT pathway, induced tumorigenesis and TRAIL resistance in NSCLC. Therefore, we hypothesized that PDGFR-α/β, through the activation of the AKT pathway should be involved in TRAIL-induced apoptosis. Indeed, overexpression of miR-34a and miR-34c or downregulation of PDGFR-α/β by siRNAs, highly increased the response of semi-resistant NSCLC cells to TRAIL-induced apoptosis. Importantly, combined treatment of a PDGFR inhibitor with TRAIL, increased apoptosis and reduced cell proliferation, as assessed by caspase 3/7 assay and MTT assays. Taken together, the results suggest that combined treatment of TRAIL with PDGFR inhibitors could sensitize a subset of lung tumors, expressing the PDGF receptors, to the drug. Moreover, it is well known that the PI3K/AKT, as well the ERK1/2 pathways regulate cellular migration and invasion of different cancers [40,41]. Here, we reported that miR-34a and miR-34c overexpression or PDGFR-α/β silencing inhibited the migration and invasion capacity of Calu-6 and H1703 cells, compared to cells transfected with a scrambled miR or siRNA control. Enforced expression of PDGFR-α or PDGFR-β partially restored NSCLC migration and invasion supporting that the regulation of the expression of these receptors by miR-34a/c plays an important role in NSCLC tumorigenesis. However, we recognize that other miR-34a/c targets including c-Met [16] and AXL [42] could also be involved. While this manuscript was in preparation Silber et al. reported that miR-34a expression was lower in proneural gliomas compared to other tumor subtypes and identified PDGFR-α as a direct target of miR-34a [43]. Here, we report that not only miR-34a but miR-34c also downregulates PDGFR-α in NSCLC cells. Moreover, we demonstrate that PDGFR-β is a miR-34a/c direct target while we did not see any significant effect on the expression of PDGFR-α and PDGFR-β after miR-34b enforced expression. Remarkably, our study shows that inhibition or downregulation of PDGFR-α and PDGFR-β by miR-34a/c antagonizes tumorigenicity and increases sensitivity to TRAIL-induced cell death with important therapeutic application for future anti-tumor therapy of lung cancer.

## Materials and Methods

### Lung cancer cell lines and tissue samples

Human H460, A549, H1299, H1703 cell lines were grown in RPMI medium containing 10% heat-inactivated fetal bovine serum (FBS) and with 2mM L-glutamine and 100Uml-1 penicillin-streptomycin. Calu-6 cells were grown in MEM supplemented with 10% fetal bovine serum, 2mM L-glutamine and 100Uml-1 penicillin–streptomycin. 9 lung tumors (including adenocarcinoma and squamous cell carcinoma) and their normal counterparts were kindly provided by Dr. S.P. Nana-Sinkam, Pulmonary, Allergy, Critical Care and Sleep Medicine, The Ohio State University Comprehensive Cancer Center, Columbus, OH. 48 lung normal and tumor tissue samples were provided from the Department of Pathology, Ohio State University. All human tissues were obtained according to a protocol approved by the Ohio State Institutional Review Board.

### Luciferase assay

The 3’ UTRs of the human PDGFRA and PDGFRB genes, were PCR amplified using the following primers:

PDGFR-α FW 5’ TCTAGACCG GCCTGAGAAACACTATTTGTG 3’
PDGFR-α RW 5’ TCTAGAACATGAACAGGGGCATTCGTAATACA 3″
PDGFR-β FW 5’ TCTAGAAAAGAGGGCAAATGAGATCACCTCCTGCA 3’
PDGFR-β RW 5’ TCTAGATATTGAGAACCCACTCTCCCTCCTTGGA 3’

and cloned downstream of the Renilla luciferase stop codon in pGL3 control vector (Promega). These constructs were used to generate, by inverse PCR, the p3’-UTRs- mutant-plasmids using the following primers:

PDGFR-α mut1 FW 5’ ACTGCCAAAACATTTATGACAAGCTGTATCGCCTCG 3’
PDGFR-α mut1 RW 5’ CGAGGCGATACAGCTTGTCATAAATGTTTTGGCAGT
PDGFR-αmut2 FW: 5’ ACTGCCAAAACATTTATGACAAGCTGTATGGTCGTTTATATTT 3’
PDGFR-αmut2 RW:5’ AAATATAAACGACCATACAGCTTGTCATAAATGTTTTGGCAGT 3’
PDGFR-β Mut FW 5'-ATGGGGGTATGGTTTTGTCAGACCTAGCAGTGAC-3'
PDGFR-β Mut RW 5'-GTCACTGCTAGGTCTGACAAAACCATACCCCCAT-3'

Calu-6 cells were cotransfected with 1μg of p3’UTR-PDGFR-α, p3’UTR-PDGFR-β or with p3’UTRmut-PDGFR-α and p3’UTRmut-PDGFR-β, 1 μg of a Renilla luciferase expression construct pRL-TK (Promega) by using Lipofectamine 2000 (Invitrogen). Cells were harvested 24h post-transfection and assayed with Dual Luciferase Assay (Promega) according to the manufacturer’s instructions. Three independent experiments were performed in triplicate.

### Western Blot Analysis

Total proteins from NSCLC were extracted with radioimmunoprecipitation assay (RIPA) buffer (0.15mM NaCl, 0.05mM Tris-HCl, pH 7.5, 1% Triton, 0.1% SDS, 0.1% sodium deoxycholate and 1% Nonidet P40). Sample extract (50 μg) was resolved on 7.5–12% SDS–polyacrylamide gels (PAGE) using a mini-gel apparatus (Bio-Rad Laboratories) and transferred to Hybond-C extra nitrocellulose. Membranes were blocked for 1h with 5% nonfat dry milk in Tris-buffered saline containing 0.05% Tween 20, incubated overnight with primary antibody, washed and incubated with secondary antibody, and visualized by chemiluminescence. The following primary antibodies were used: anti-PDGFR-α, anti-PDGFR-β, anti-ERK1/2, anti-p-ERKs, anti-pAKT, anti-total AKT, anti-GAPDH antibodies (Cell Signaling). A secondary anti-rabbit or anti-mouse immunoglobulin G (IgG) antibody peroxidase conjugate (Chemicon) was used.

### Real-time PCR

Real-time PCR was performed using a standard TaqMan PCR Kit protocol on an Applied Biosystems 7900HT Sequence Detection System (Applied Biosystems). The 10 μl PCR reaction included 0.67 μl RT product, 1 μl TaqMan Universal PCR Master Mix (Applied Biosystems), 0.2 mM TaqMan probe,1.5 mM forward primer and 0.7 mM reverse primer. The reactions were incubated in a 96-well plate at 95 ^°^C for 10 min, followed by 40 cycles of 95 ^°^C for 15 s and 60 ^°^C for 1 min. All reactions were run in triplicate. The threshold cycle (CT) is defined as the fractional cycle number at which the fluorescence passes the fixed threshold. The comparative CT method for relative quantization of gene expression (Applied Biosystems) was used to determine miRNA expression levels. The y axis represents the 2(-ΔCT), or the relative expression of the different miRs. MiRs expression was calculated relative to U44 and U48 rRNA. Experiments were carried out in triplicate for each data point, and data analysis was performed by using software (Bio- Rad).

### Cell death and cell proliferation quantification

For detection of caspase 3/7 activity, cells were cultured in 96-well plates, in triplicate, treated with TRAIL (100-150ng/ml) and analyzed using Caspase-Glo 3/7 Assay kit (Promega) according to the manufacturer’s instructions. Continuous variables are expressed as mean values ± standard deviation (s.d.). Cell viability was examined with 3- (4,5-dimethylthiazol-2-yl)-2,5-dipheniltetrazolium bromide (MTT)-Cell Titer 96 AQueous One Solution Cell Proliferation Assay (Promega), according to the manufacturer’s protocol. Metabolically active cells were detected by adding 20 μl of MTT to each well. After 1 h of incubation, the plates were analyzed in a Multilabel Counter (Bio-Rad Laboratories).

### Anti-PDGFR-α and anti-PDGFR-β siRNAs transfection

Cells were cultured to 50% confluence and transiently transfected for 72h using Lipofectamine 2000 with 100 nM anti-PDGFR-α and/or with 100nM anti-PDGFR-β SMARTpool siRNAs or control siRNAs (Dharmacon), a pool of four target specific 20–25 nt siRNAs designed to knock down gene expression.

### MiRNA locked nucleic acid in situ hybridization of formalin fixed, paraffin-embedded tissue section

In situ hybridization (ISH) was carried out on deparaffinized human lung tissues using previously published protocol[44], which includes a digestion in pepsin (1.3 mg/ml) for 30 minutes. The sequence of the probe containing the dispersed locked nucleic acid (LNA) modified bases with digoxigenin conjugated to the 5’ end was: 5’ ACAACCAGCTAAGACACTGCCA 3’. The probe cocktail and tissue miRNA were co-denatured at 60 ^°^C for 5 minutes, followed by hybridization at 37 ^°^C overnight and a stringency wash in 0.2X SSC and 2% bovine serum albumin at 4 ^°^C for 10 minutes. The probe-target complex was seen due to the action of alkaline phosphatase on the chromogen nitroblue tetrazolium and bromochloroindolyl phosphate (NBT/BCIP). Negative controls included the use of a probe, which should yield a negative result in such tissues (scrambled miRNA). No counterstain was used, to facilitate co-labeling for PDGFR-α and PDGFR-β protein. After in situ hybridization for the miRNAs, as previously described (Nuovo et al., 2009), the slides were analyzed for immunohistochemistry using the optimal conditions for PDGFR-α (1:100, cell conditioning for 30 minutes) and PDGFR-β (1:200, cell conditioning for 30 minutes). For the immunohistochemistry, we used the Ultrasensitive Universal Fast Red or DAB systems from Ventana Medical Systems. The percentage of tumor cells expressing PDGFR-α, PDGFR-β and miR-34a, was then analyzed with emphasis on co-localization of the respective targets. Co-expression analysis was done with the Nuance system (Cambridge Research Institute) per the manufacturer’s recommendations.

### Bioinformatics analysis

Bioinformatics analysis was performed by using these specific programs:

Targetscan1, Pictar2, RNhybrid 3:

1 http://www.targetscan.org/
2 http://pictar.bio.nyu.edu/
3 http://bibiserv.techfak.uni-bielefeld.de/

### PDGFR-α and PDGFR-β plasmids

cDNA-PDGFR-α (Cat. Number MHS1010-9205933) and cDNA-PDGFR-β (Cat. MHS1010-7430189) were purchased from Open Biosystems. H460 TRAIL-sensitive cells were transfected with 1μg of each plasmid and proliferation and caspase 3/7 assays were performed as previously described.

### Migration assay

Briefly, Calu-6 cells were transfected with pcDNA-PDGFR-α, pcDNA-PDGFR-β and/or hsa-miR-34a and miR-34c, respectively. 24h after transfection, 2x10^5^ cells in MEM media supplemented with 1% FBS were plated into the upper chambers of the Migration assay and RPMI supplemented with 10% FBS were added into lower chambers to use as a chemoattractant. After 24h, the upper chambers were transferred into a new plate with detaching solutionscontaining Calcein AM for 1 hour to measure the amount of the migrated cells. The fluorescence was analyzed at an excitation wavelength of 485 nm and an emission wavelength of 520nm.

### Invasion assay

Briefly, 2x 10^5^ Calu-6 cells transfected with pcDNA-PDGFR-α, pcDNA-PDGFR-β and/or miR-34a and miR-34c in RPMI supplemented with 1% FBS were plated into upper chambers of Invasion assay with a 8-um pore size-polycarbonate membrane. 700 μl of MEM supplemented with 10% FBS were added into the lower chambers as a chemoattractant. After 36-48h, the upper chambers were transferred into a new plate and were incubated with detaching solutions contained Calcein AM for 1 hour to measure the amount of the invaded cells. The fluorescence was analyzed at an excitation wavelength of 485 nm and an emission wavelength of 520nm.

### Statistical analysis

Student’s t test was used to determine significance. All error bars represent the standard error of the mean. Statistical significance for all the tests, assessed by calculating P-value, was < 0.05.

## Supporting Information

File S1 Figure S1, NSCLC cell lines analyzed and p53 status. (**a**) A panel of 5 NSCLC cells with their p53 status is reported. (**b**) qRT-PCR showing low expression of miR-34a,-34b,-34c in 5 different NSCLC cells. Figure S2, **Co-expression analysis of miR-34a** and **PDGFR-α** and **PDGFR-β in lung tumor samples**. Tables reporting the percentage of miR-34a, PDGFR-α and PDGFR-β expression observed in the 106 (PDGFR-α) and 107 (PDGFR-β) tumor samples analyzed (A case with 10% of the tumor cells + was scored as +). Figure S3, Enforced expression of **miR-34a** and **miR-34c** or PDGFR-α**/**β silencing increases the response to TRAIL-induced apoptosis and reduces tumorigenicity of NSCLC cell. (**a**) Proliferation assay showing that miR-34a and -34c enforced expression in Calu-6 and H1703 cells increases the response to TRAIL-induced apoptosis. (**b**) MTT assay showing that PDGFR-α or PDGFR-β silencing increases the response to TRAIL-induced apoptosis. (**c**) PDGFR-α or PDGFR-β overexpression in H460 TRAIL-sensitive cells decreases the response to the drug as assessed by caspase 3/7 activity. (**d**) Combined treatment of PDGFR inhibitor (20 μM) and TRAIL for 24h sensitizes NSCLC cells to TRAIL-induced apoptosis. * *P*< 0.05. Figure S4, **PDGFR-α** or **PDGFR-β overexpression reduces the response to TRAIL-induced apoptosis**. (**a**) Proliferation assay showing that miR-34a/c increase the response to TRAIL-induced apoptosis. Co-transfection of miR-34a/c with PDGFR-α/β significantly decreases the response to the drug. (**b**) PDGFR-α/β enforced expression along with miR-34a/c reduce the response to TRAIL-induced apoptosis as assessed by caspase 3/7 assay. * *P*< 0.05.
(PDF)
