# Correction: MiR-34a/c-Dependent PDGFR-α/β Downregulation Inhibits Tumorigenesis and Enhances TRAIL-Induced Apoptosis in Lung Cancer

**DOI:** 10.1371/journal.pone.0131729

**Published:** 2015-06-25

**Authors:** Michela Garofalo, Young-Jun Jeon, Gerard J. Nuovo, Justin Middleton, Paola Secchiero, Pooja Joshi, Hansjuerg Alder, Natalya Nazaryan, Gianpiero Di Leva, Giulia Romano, Melissa Crawford, Patrick Nana-Sinkam, Carlo M. Croce

After the publication of the article, it was noticed that fragments of text in this article overlap with that from previous publications. The overlap in the text relates to the Introduction, Results and Discussion sections, where sentences were reproduced without quotation marks. We would like to acknowledge this and include the relevant references. It should be noted that no concerns have been raised regarding the originality of the work reported in the article and that this has no bearing on the results and conclusions of the study.

In the Introduction section there is some overlap in text with that from the following publications:

McDermott U, Ames RY, Iafrate AJ, Maheswaran S, Stubbs H, Greninger P, et al. Ligand-dependent platelet-derived growth factor receptor (PDGFR)-alpha activation sensitizes rare lung cancer and sarcoma cells to PDGFR kinase inhibitors. Cancer Res. 2009 May 1;69(9):3937–46. doi: 10.1158/0008-5472.CAN-08-432

Hermeking H. The miR-34 family in cancer and apoptosis. Cell Death Differ. 2010 Feb;17(2):193–9. doi: 10.1038/cdd.2009.56.

Raica M, and Cimpean AM. Platelet-Derived Growth Factor (PDGF)/PDGF Receptors (PDGFR) Axis as Target for Antitumor and Antiangiogenic Therapy. Pharmaceuticals (Basel). 2010 Mar; 3(3): 572–599. doi: 10.3390/ph3030572

In the Results section there is some overlap in text with that from the following article:

Tanaka N, Toyooka S, Soh J, Kubo T, Yamamoto H, Maki Y, et al. Frequent methylation and oncogenic role of microRNA-34b/c in small-cell lung cancer. Lung Cancer. 2012 Apr;76(1):32–8. doi: 10.1016/j.lungcan.2011.10.002.

In the Discussion section there is some overlap in text with that from the following publications:

Zhang H, Bajraszewski N, Wu E, Wang H, Moseman AP, Dabora SL, Griffin JD, and Kwiatkowski DJ. PDGFRs are critical for PI3K/Akt activation and negatively regulated by mTOR. J Clin Invest. 2007 Mar;117(3):730-8.doi:10.1172/JCI28984.

West KA, Castillo SS and Dennis PA. Activation of the PI3K/Akt pathway and chemotherapeutic resistance. Drug Resist Updat. 2002 Dec;5(6):234–48
