# Correction: MiR-34a/c-Dependent PDGFR-α/β Downregulation Inhibits Tumorigenesis and Enhances TRAIL-Induced Apoptosis in Lung Cancer

**DOI:** 10.1371/journal.pone.0267628

**Published:** 2022-04-20

**Authors:** Michela Garofalo, Young-Jun Jeon, Gerard J. Nuovo, Justin Middleton, Paola Secchiero, Pooja Joshi, Hansjuerg Alder, Natalya Nazaryan, Gianpiero Di Leva, Giulia Romano, Melissa Crawford, Patrick Nana-Sinkam, Carlo M. Croce

After publication of this article [1] and initial correction [2], The Ohio State University informed *PLOS ONE* of the outcome of an investigation into this article. This investigation concluded that the article contains additional instances of text overlap that were not acknowledged in the previous correction [2].

We would like to acknowledge this and include the relevant references. It should be noted that no concerns have been raised regarding the originality of the work reported in the article, and that this has no bearing on the results and conclusions of the study.

In the Introduction section, there is some overlap in text with that from Bauman et al. (2007) [3] and Gallardo et al. (2009) [4].

The *PLOS ONE* Editors also identified instances where text from some of the authors’ own previously published work was reused in [1], as follows.

In the Results and Methods sections, there is some overlap in text with Acunzo et al. (2012) [5].

In the Methods section, there is some overlap in text with Garofalo et al. (2012) [6] and Garofalo et al. (2008) [7].

In the legend for Figure 1, there is some overlap in text with Garofalo et al. (2009) [8].
