# Supplementary material for: MiR-34a/c-Dependent PDGFR-α/β Downregulation Inhibits Tumorigenesis and Enhances TRAIL-Induced Apoptosis in Lung Cancer
Source: PLoS One. 2013 Jun 21;8(6):e67581. doi: 10.1371/journal.pone.0067581 (PMC3689725; doi:10.1371/journal.pone.0067581)
Supplement: File S1 — (a) A panel of 5 NSCLC cells with their p53 status is reported. (b) qRT-PCR showing low expression of miR-34a,-34b,-34c in 5 different NSCLC cells. Figure S2, Co-expression analysis of miR-34a and PDGFR-α and PDGFR-β in lung tumor samples. Tables reporting the percentage of miR-34a, PDGFR-α and PDGFR-β expression observed in the 106 (PDGFR-α) and 107 (PDGFR-β) tumor samples analyzed (A case with 10% of the tumor cells + was scored as +). Figure S3, Enforced expression of miR-34a and miR-34c or PDGFR-α/β silencing increases the response to TRAIL-induced apoptosis and reduces tumorigenicity of NSCLC cell. (a) Proliferation assay showing that miR-34a and -34c enforced expression in Calu-6 and H1703 cells increases the response to TRAIL-induced apoptosis. (b) MTT assay showing that PDGFR-α or PDGFR-β silencing increases the response to TRAIL-induced apoptosis. (c) PDGFR-α or PDGFR-β overexpression in H460 TRAIL-sensitive cells decreases the response to the drug as assessed by caspase 3/7 activity. (d) Combined treatment of PDGFR inhibitor (20 μM) and TRAIL for 24h sensitizes NSCLC cells to TRAIL-induced apoptosis. * P< 0.05. Figure S4, PDGFR-α or PDGFR-β overexpression reduces the response to TRAIL-induced apoptosis. (a) Proliferation assay showing that miR-34a/c increase the response to TRAIL-induced apoptosis. Co-transfection of miR-34a/c with PDGFR-α/β significantly decreases the response to the drug. (b) PDGFR-α/β enforced expression along with miR-34a/c reduce the response to TRAIL-induced apoptosis as assessed by caspase 3/7 assay. * P< 0.05. (PDF) [file pone.0067581.s001.pdf]

**a**

| Cell lines | P53 status |
|---|---|
| H1299 | null |
| A549 | WT |
| Calu-6 | null |
| H1703 | mutated |
| H460 | WT |

**b**

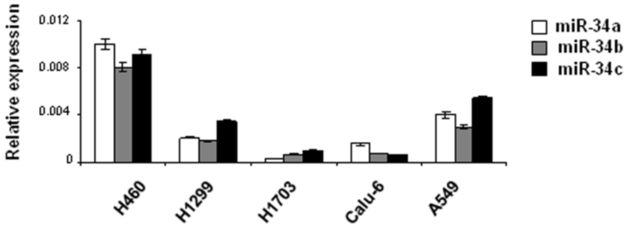


**Figure S1**

**miR-34a and PDGFR alpha co-expression analyses**

| miR34+/PDGFRα+ | PDGFRα-/miR-34a+ | PDGFRα+/ miR-34a- | Each negative |
|---|---|---|---|
| 9/106 (8%) | 9/106 (8%) | 40/106 (38%) | 48/106 (46%) |

**miR-34a and PDGFR beta co-expression analyses**

| miR34+/PDGFRβ+ | PDGFRβ-/miR-34a+ | PDGFRβ+/miR-34a- | Each negative |
|---|---|---|---|
| 9/107 (8%) | 10/107 (9%) | 34/107 (32%) | 54/107 (51%) |

**Figure S2**

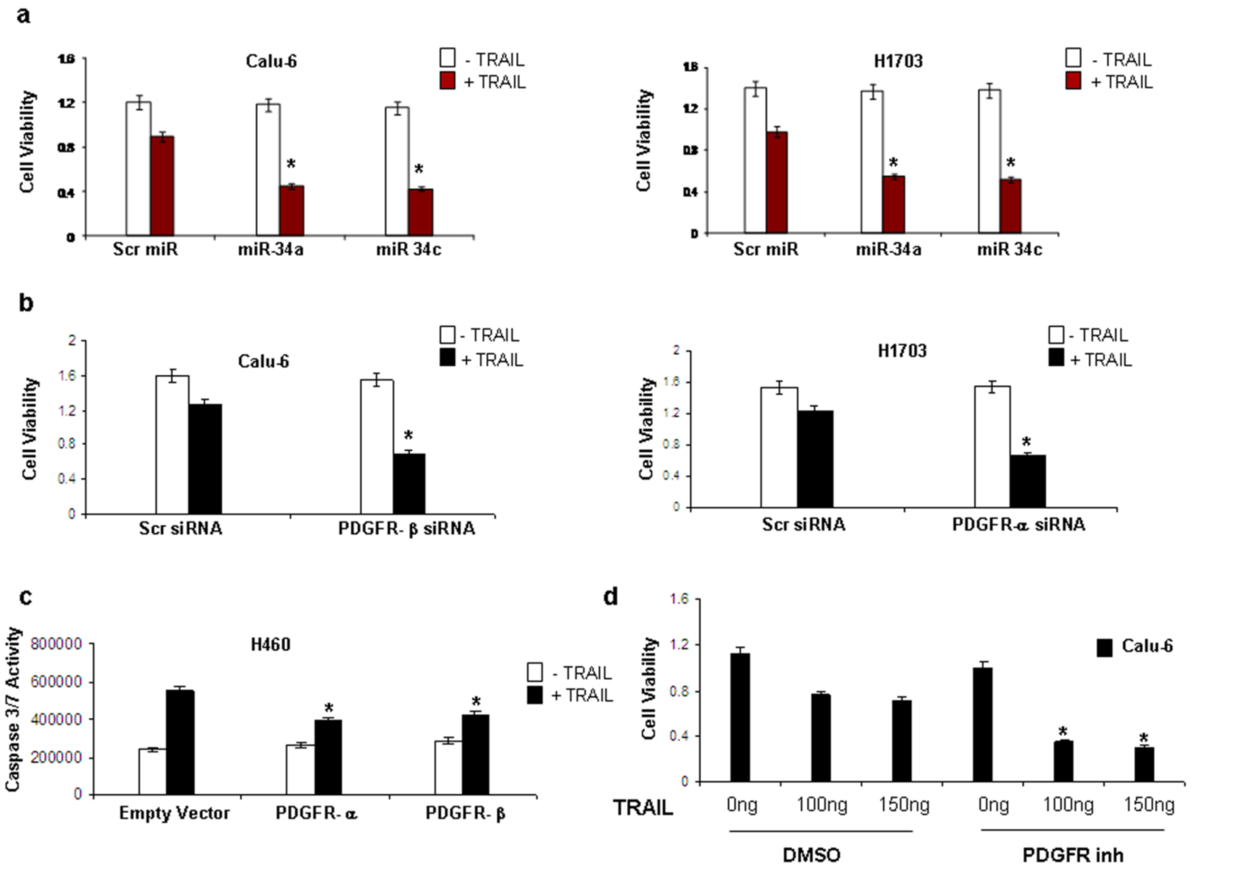
a
Calu-6
H1703
- TRAIL
+ TRAIL
Cell Viability
Scr miR
miR-34a
miR 34c
b
Calu-6
H1703
Scr siRNA
PDGFR- β siRNA
PDGFR-α siRNA
c
H460
Caspase 3/7 Activity
Empty Vector
PDGFR- α
PDGFR- β
d
Calu-6
TRAIL
0ng
100ng
150ng
DMSO
PDGFR inh

Figure S3

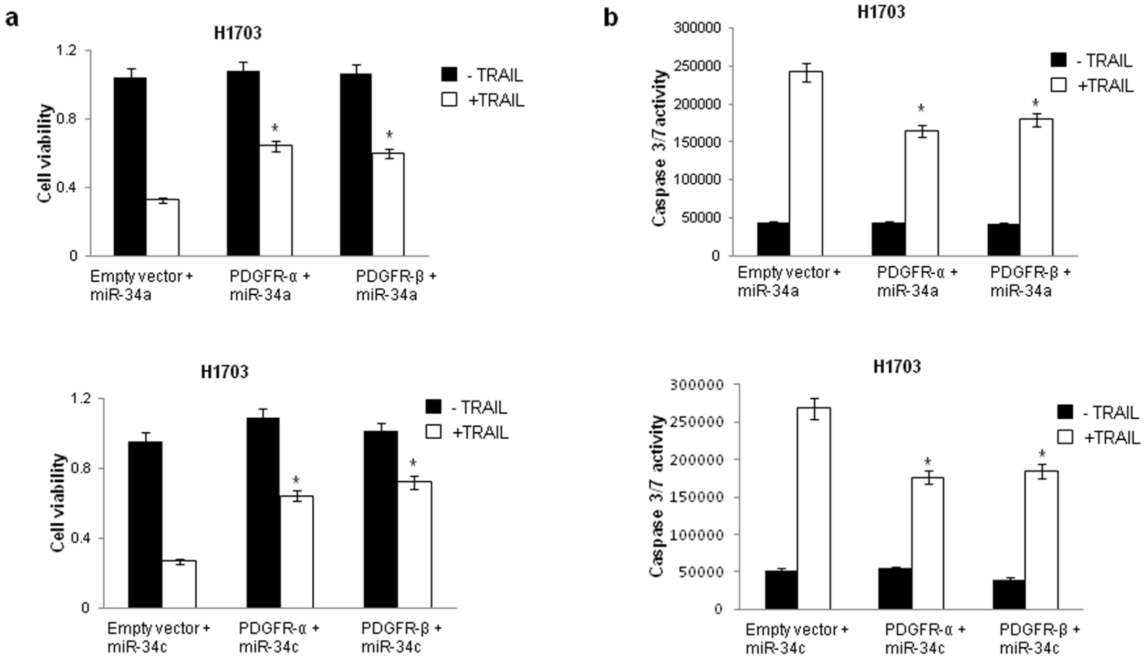
a
H1703
Cell viability
1.2
0.8
0.4
0
- TRAIL
+TRAIL
Empty vector + miR-34a
PDGFR-α + miR-34a
PDGFR-β + miR-34a
H1703
Cell viability
Empty vector + miR-34c
PDGFR-α + miR-34c
PDGFR-β + miR-34c
b
H1703
Caspase 3/7 activity
300000
250000
200000
150000
100000
50000
0
- TRAIL
+TRAIL
Empty vector + miR-34a
PDGFR-α + miR-34a
PDGFR-β + miR-34a
H1703
Caspase 3/7 activity
Empty vector + miR-34c
PDGFR-α + miR-34c
PDGFR-β + miR-34c

**Figure S4**
